# Contribution of 32 GWAS-Identified Common Variants to Severe Obesity in European Adults Referred for Bariatric Surgery

**DOI:** 10.1371/journal.pone.0070735

**Published:** 2013-08-07

**Authors:** Reedik Mägi, Sean Manning, Ahmed Yousseif, Andrea Pucci, Ferruccio Santini, Efthimia Karra, Giorgia Querci, Caterina Pelosini, Mark I. McCarthy, Cecilia M. Lindgren, Rachel L. Batterham

**Affiliations:** 1 Wellcome Trust Centre for Human Genetics, University of Oxford, Oxford, United Kingdom; 2 Estonian Genome Center, University of Tartu, Tartu, Estonia; 3 Centre for Obesity Research, Rayne Institute, Department of Medicine, University College London, London, United Kingdom; 4 University College London Hospitals/University College London National Institute of Health Research Biomedical Research Centre, London, United Kingdom; 5 University College London Hospitals Centre for Weight loss, Metabolic and Endocrine Surgery, University College London Hospitals, London, United Kingdom; 6 Obesity Center at the Endocrinology Unit, University Hospital of Pisa, Pisa, Italy; 7 Oxford Centre for Diabetes, Endocrinology and Metabolism, University of Oxford, Oxford, United Kingdom; 8 Oxford National Institute of Health Research Biomedical Research Centre, Churchill Hospital, Oxford, United Kingdom; The Children’s Hospital of Philadelphia, United States of America

## Abstract

The prevalence of severe obesity, defined as body mass index (BMI) ≥35.0 kg/m^2^, is rising rapidly. Given the disproportionately high health burden and healthcare costs associated with this condition, understanding the underlying aetiology, including predisposing genetic factors, is a biomedical research priority. Previous studies have suggested that severe obesity represents an extreme tail of the population BMI variation, reflecting shared genetic factors operating across the spectrum. Here, we sought to determine whether a panel of 32 known common obesity-susceptibility variants contribute to severe obesity in patients (n = 1,003, mean BMI 48.4±8.1 kg/m^2^) attending bariatric surgery clinics in two European centres. We examined the effects of these 32 common variants on obesity risk and BMI, both as individual markers and in combination as a genetic risk score, in a comparison with normal-weight controls (n = 1,809, BMI 18.0–24.9 kg/m^2^); an approach which, to our knowledge, has not been previously undertaken in the setting of a bariatric clinic. We found strong associations with severe obesity for SNP rs9939609 within the *FTO* gene (*P = *9.3×10^−8^) and SNP rs2815752 near the *NEGR1* gene (*P = *3.6×10^−4^), and directionally consistent nominal associations (*P*<0.05) for 12 other SNPs. The genetic risk score associated with severe obesity (*P = *8.3×10^−11^) but, within the bariatric cohort, this score did not associate with BMI itself (*P = *0.264). Our results show significant effects of individual BMI-associated common variants within a relatively small sample size of bariatric patients. Furthermore, the burden of such low-penetrant risk alleles contributes to severe obesity in this population. Our findings support that severe obesity observed in bariatric patients represents an extreme tail of the population BMI variation. Moreover, future genetic studies focused on bariatric patients may provide valuable insights into the pathogenesis of obesity at a population level.

## Introduction

Obesity is a serious and increasing threat to the health of populations globally. The high burden of obesity-related co-morbidities, such as type 2 diabetes, cardiovascular disease and certain cancers, heighten the severity of this obesity crisis. Globally, in 2008 over 200 million men and almost 300 million women were obese, defined by body mass index (BMI ≥30 kg/m^2^), which represents an approximate doubling of the prevalence of obesity since 1980 [1]. Alarmingly, the prevalence of severe obesity (BMI ≥35 kg/m^2^) continue to rise rapidly in westernised societies [2], [3], [4], despite a flattening in the trend for levels of overall obesity [5]. Given the disproportionately higher health burden and healthcare costs associated with severe obesity [6], understanding the underlying mechanisms, including genetic factors, is a biomedical research priority.

BMI is heavily dependent on genetic susceptibility as demonstrated by twin and adoption studies [7], [8]. Furthermore, in the quest to elucidate the biological basis of obesity, genome wide association studies (GWAS) using single nucleotide polymorphisms (SNPs) have identified more than 40 genetic variants to date that are associated with BMI or risk for obesity (defined as BMI ≥30 kg/m^2^) [9], . The finding that multiple common variants have effects on the risk of being obese invokes the ‘common disease, common variant’ hypothesis of obesity [12], [13]. However, the considerable gap between the 2% of variance attributable to common variants and the much higher estimated heritability raises questions over what constitutes the genetic basis of obesity [12], [13]. Previous studies have suggested that individuals who have extreme obese phenotypes, such as early onset (BMI ≥95th percentile achieved before the age of 10–18 years old) [14], [15] or severe adult obesity (BMI ≥35.0 kg/m^2^) [16], represent an extreme tail of the population BMI variation, with a higher burden of shared genetic factors [17]. Alternatively, extreme obesity may be viewed as a separate entity with distinct underlying genetic factors [18]. Previous GWAS reports for severe adult obesity case-control samples identified associations only with SNPS within the intronic *FTO* locus [16], [19], consistent with the robust association of these SNPs with BMI in the general population [10]. In contrast, other GWAS evaluating early onset extreme obesity detected loci distinct from those identified in the meta-analysis of adult BMI [20], [21], [22]. Importantly, a recent genome-wide analysis for loci associated with clinical classes of obesity and extreme BMI tails [11], drawn from populations within the prior Genetic Investigation of ANthropometric Traits (GIANT) meta-analysis of adult BMI, detected no new loci associated with class 3 obesity (BMI ≥40 kg/m^2^), in addition to those uncovered in the original meta-analysis. While two new loci were found to be associated with class 2 obesity (BMI 35.0–39.9 kg/m^2^), this study provides strong evidence that the majority of common BMI-increasing variants continue to have effects across the full BMI spectrum.

Bariatric or weight-loss surgery is indicated for patients with a BMI ≥40.0 kg/m^2^ or ≥35.0 kg/m^2^ in the presence of at least one obesity-related comorbidity, and currently represents the most effective treatment for patients with severe obesity [23]. In light of the marked health benefits of bariatric surgery, the number of patients being referred for bariatric surgery assessment is increasing, with over 340,000 bariatric procedures undertaken in 2011 [24]. The assessment of patients in bariatric surgery centres thus offers an opportunity to undertake genetic studies in a population at the upper tail of the BMI spectrum.

Therefore, we investigated whether 32 known common obesity-susceptibility variants are enriched in a cohort of patients with severe obesity attending a bariatric surgical assessment clinic, compared with normal-weight (BMI 18.0–24.9 kg/m^2^) controls. We found that a genetic risk score, calculated based on all 32 genotyped SNPs, is associated with severe obesity but there was no significant effect of the genetic risk score on BMI within the bariatric cohort.

## Methods

### Study Population and Anthropometric Measures

Patients were recruited from two bariatric centres; the University College London Hospitals (UCLH) Centre for Weight-loss, Metabolic and Endocrine Surgery, London, UK and the University Hospital of Pisa (UHP), Pisa, Italy. Individuals of European descent were included in the analyses in order to facilitate comparison with a UK population-based control group. Patients with a BMI ≥35.0 kg/m^2^ who donated a peripheral blood sample for DNA analysis were recruited to the study. At the UCLH Centre, 585 patients who attended bariatric surgery clinics were recruited between October 2009 and October 2012. Of these 585 patients, 26 were excluded due to incomplete clinical data (n = 11), absence of genotyping (n = 6), unsuccessful genotyping (n = 4) or a documented BMI of <35.0 kg/m^2^ (n = 5), which resulted in a total of 559 patients from the UCLH centre being included. 36 UCLH patients, who had undergone previous bariatric surgery for treatment of severe obesity (BMI ≥35.0 kg/m^2^) at other bariatric centres, were included in the case-control analysis but not in the analysis for SNP effects on BMI. 444 Italian patients who attended to the Obesity Centre at the Endocrinology Unit of the University Hospital of Pisa, Italy, from January 2003 to December 2011, for evaluation prior to bariatric surgery, were recruited to the study. Thus, 1,003 samples were available for the case-control analysis and 967 (excluding n = 36 patients who had previous bariatric surgery) were included in the within-group analysis. BMI was calculated from the weight and height measurements recorded at the first visit to the bariatric clinic. Weight was measured using the Walkthrough Platform A12SS Stainless Steel Indicator. Height was measured using a wall-mounted digital stadiometer. Demographic and comorbidity data were collected by means of an electronic clinical data record. The National Health Service Research Ethics Committee approved the research protocol (ID#09/H0715/65) and all participants provided written informed consent. The control group was comprised of normal-weight population-based controls from the British 1958 Birth Cohort (B58C) who were previously genotyped either as part of the Wellcome Trust Case Control Consortium 2 [25] or another related genotyping effort [26]. From a total of 5,382 B58C reference samples, we selected individuals with BMI in the normal range (18.0–24.9 kg/m^2^, mean 22.8±1.6 kg/m^2^), amounting to 1,809 normal-weight controls, 64% of whom were female and 36% male. B58C controls had anthropometric data measured during a biomedical examination undertaken at the age of 44–45 years [27].

### DNA Extraction and Quantification of Bariatric Surgery Case Samples

All DNA extractions from peripheral blood samples were performed using the QIAamp DNA Blood Midi Kit (Qiagen) according to the manufacturer’s instructions. DNA concentration and purity were determined with UV spectrophotometry (Nanodrop) measuring the spectrophotometric absorbance ratios of 260 nm/280 nm. High quality DNA was considered to have an A_260_/A_280_ ratio of 1.85 - 2.10. All genomic DNA was diluted to a final concentration of 5 ng/µl.

### SNP Genotyping of Bariatric Surgery Case Samples

30 single nucleotide polymorphisms (SNPs) corresponding to loci identified in the GIANT meta-analysis of adult BMI [10] were genotyped. Genotyping was not successful for two other SNPs, rs12444979 near *GPRC5B* and rs4836133 near *ZNF608*. Two further SNPs, corresponding to the additional loci near *HOXB5* and *OLFM4* uncovered in a meta-analysis of childhood severe obesity [14] and which also yielded directionally consistent associations in the meta-analysis of adult BMI [10], were also genotyped. Of note, the *FTO* SNP genotyped was rs9939609, which was the SNP reported in the first GIANT GWAS [9] and is in strong to complete linkage disequilibrium with other reported intronic *FTO* SNPs [28]. Genotyping of bariatric patients was performed by KBioscience (Hertfordshire, UK). SNPs were genotyped using the KASP (KBioscience Competitive Allele-Specific PCR) SNP genotyping system (www.lgcgenomics.com/genotyping/kasp-genotyping-reagents/). The following quality criteria were applied to both bariatric cases and B58C control samples: HWE p-value >0.0001, genotype callrate >95%, and sample callrate >90%. Blind duplicates were used to detect possible DNA mixup.

### Statistical Analysis

Statistical analyses were performed using the programs PLINK [29], SNPTEST [30], and R software environment [31]. Logistic regression analyses were performed using an additive genetic model to evaluate the difference between the normal-weight control group (n = 1,809) and the sample of bariatric surgery patients (n = 1,003). Additionally, linear regression analyses with an additive genetic model were performed for BMI within the bariatric sample-set alone (n = 967, excluding n = 36 patients who had previous bariatric surgery), firstly using standardized BMI values (see Model S1 in File S1 for standardization formula) in order to compare effect sizes within the bariatric cohort with the known effect sizes derived from inverse standardized BMI values in the published meta-analysis [10], and secondly, using unstandardized BMI values in considering the BMI distribution of the bariatric cohort sample-set. To compare between the reference effect sizes from the published meta-analysis [10] and effect sizes in the bariatric cohort, we used a standard t-test. Secondary logistic and linear regression analyses were performed using both dominant and recessive models. Power analysis for single marker effects, performed with a genetic power calculator (http://pngu.mgh.harvard.edu/~purcell/cgi-bin/cc2k.cgi) taking a trait prevalence of 4%, a risk allele frequency of 20%, and p-value threshold adjusted for 32 independent samples (α = 0.05/32 = 0.00132), showed that power estimates for genotype relative risks of 1.1, 1.15, 1.2 and 1.25 were 4.2%, 15.1%, 36.9% and 62.9% respectively. All analyses were adjusted for gender, age and country of origin. A previous analysis demonstrated that the common BMI-increasing SNPs do not appear to have strong allele frequency differences across five diverse European populations, including the B58C cohort and an Italian cohort [10], therefore a specific Italian control sample-set was not sought.

Multiple marker analyses were performed with PLINK [29] using genetic scores calculated from all 32 genotyped markers with their relative weight based on their effect sizes in the published meta-analysis [10] (Model S2 in File S1). Linear regression model in R was used for evaluating the predictive value of the genetic score in relation to BMI within the bariatric cohort and logistic regression was used to determine the extent to which genetic scores distinguished between the normal-weight control and bariatric cohort groups.

## Results

**Figure 1:**
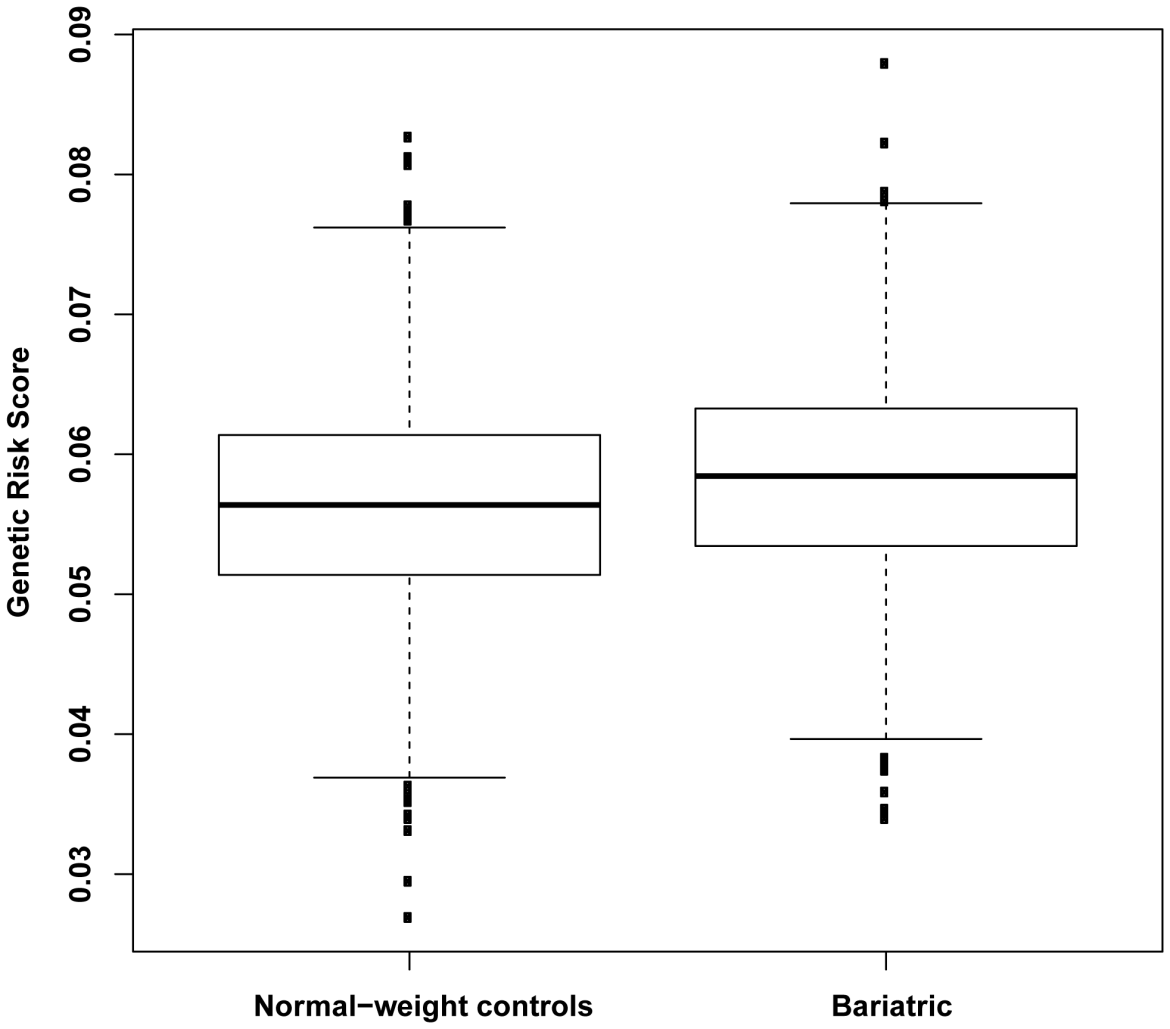
The boxplot displays genetic risk scores in bariatric patients compared to normal-weight controls. The average genetic risk score differentiated well between normal-weight controls group and the bariatric surgery group *(P* = 8.3×10^−11^).

**Figure 2:**
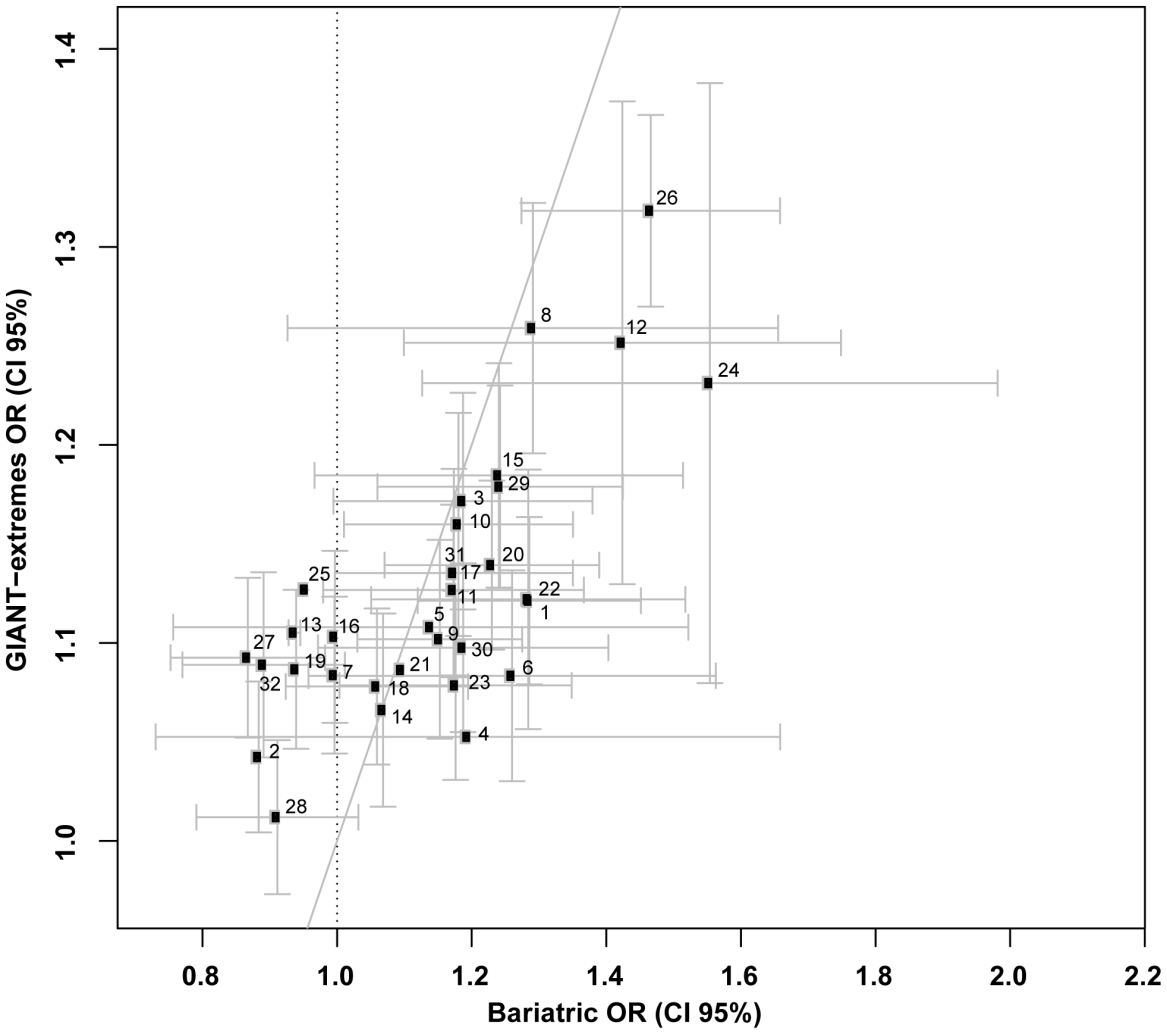
Results of our logistic regression analysis were compared with the GIANT-extremes results using combined data from obesity class 2 and 3 groups [11]; in terms of odds ratio (OR) with 95% confidence intervals (CI). There were no significant differences between the compared OR. See Table 2 for allocated reference numbers of SNPs. The diagonal line represents the expected plotted values for our results, based on the GIANT-extremes results. The SNPs below the diagonal line are those which had a larger effect in our study compared to GIANT-extremes, whereas the SNPs above the diagonal line represent SNPs which had a larger effect in GIANT-extremes compared to our study.

A total of 1,003 patients attending a bariatric surgery assessment clinic were included in the case-control analyses (see Table 1 for baseline demographic and anthropometric characteristics). Firstly, we undertook a comparison of the effects of BMI-raising SNPs in the normal-weight control (n = 1,809) and the bariatric surgery (n = 1,003) groups to determine whether known BMI-increasing SNPs are associated with severe obesity in our cohort. We found associations for SNP rs9939609 within the *FTO* gene (*P* = 9.3×10^−8^) and SNP rs2815752 near the *NEGR1* gene (*P = *3.6×10^−4^). Directionally consistent nominal associations were also detected for SNPs at the *FAIM2, TMEM18, PRKD1* and *MC4R(B)* loci (*P*<0.01), and at the *SLC39A8, TNNI3K*, *OLFM4, LRP1B, KCTD15*, *TFAP2B, GNPDA2* and *SEC16B* loci (*P*<0.05) (Table 2). Analysis of SNPs at the other 18 loci did not reveal any evidence of association. However, 9 of these 18 SNPs had effects directionally consistent with the GIANT meta-analysis results (Table 2). Secondary analyses using both dominant and recessive models revealed similar results to the additive model (Table S1 in File S1). Stronger associations were found for six SNPs using the dominant model and for two SNPs using the recessive model (Table S1 in File S1). Upon combining all 32 genotyped SNPs into a genetic risk score, we found a significant difference in the average risk score between normal-weight control group and the bariatric surgery group (P = 8.3×10^−11^, adjusted R^2^ = 0.0043) (Figure 1). Comparison of the effects of BMI-associated SNPs in patients in specific BMI categories in the bariatric cohort (<40.0 kg/m^2^, 40.0–44.9 kg/m^2^, 45.0–49.9 kg/m^2^, 50.0–59.9 kg/m^2^, ≥60.0 kg/m^2^) and the normal-weight control group revealed that both the strongest effects for the SNP rs9939609 within the *FTO* gene (*β = *1.08±0.23, *P = *3.4×10^−6^) and the weakest effects for the SNP rs2815752 near the *NEGR1* gene (*β = *0.02±0.22, *P = *0.9) were in the ≥60.0 kg/m^2^ BMI category (Table 3)**.** In order to place our findings in the context of the recent GIANT-extremes results [11], we compared the effects of the BMI-increasing SNPs on odds of severe obesity in our study with those from the GIANT-extremes analyses and detected no significant differences (Figure 2).

**Table 1:** Baseline demographic and clinical characteristics of bariatric patients.

	All	UCLH	UHP
**Total number (n)**	1,029	585	444
**Excluded (n, %)**	26	26	0
**Included (n, %)**	1,003	559 (56)	444 (44)
**Age** * **(years)**	44.6±11	45.5±10.8	43.5±11.1
**Female (%)**	709 (71)	370 (66)	339 (76)
**Male (%)**	294 (29)	189 (34)	105 (24)
**BMI** * **(kg/m^2^)**	48.4±8.1	48.7±7.9	48.2±8.3
**Type 2 diabetes (n, %)**	260 (26)	157 (28)	104 (23)
**Metabolic risk** ** **(n, %)**	583 (58)	299 (53)	284 (64)
**Prev. bariatric surgery (n, %)**	36 (4)	36 (6)	0 (0)

* Data are shown as mean ± SD.

** Defined as presence of ≥1 major cardiovascular risk factor.

**Table 2:** Results of logistic regression for the 32 genotyped SNPs.

#Ref	Nearest gene	Chr	rsid	EA	EAF cases	EAF controls	β	*P*	OR	
**#1**	***NEGR1***	1	rs2815752	A	0.67	0.59	0.25	3.6×10^−4^		**1.29**
**#2**	***PTBP2***	1	rs1555543	C	0.57	0.60	−0.12	0.08		*0.88*
**#3**	***SEC16B***	1	rs543874	G	0.22	0.21	0.17	0.04		**1.19**
**#4**	***TNNI3K***	1	rs1514175	A	0.45	0.41	0.18	0.01		**1.19**
**#5**	***FANCL***	2	rs887912	T	0.32	0.29	0.13	0.09		1.14
**#6**	***LRP1B***	2	rs2890652	C	0.16	0.14	0.23	0.02		**1.26**
**#7**	***RBJ***	2	rs713586	C	0.48	0.49	−0.004	0.95		*0.996*
**#8**	***TMEM18***	2	rs2867125	C	0.84	0.81	0.26	0.005		**1.29**
**#9**	***CADM2***	3	rs13078807	G	0.22	0.19	0.14	0.11		1.15
**#10**	***ETV5***	3	rs9816226	T	0.83	0.81	0.17	0.07		1.18
**#11**	***GNPDA2***	4	rs10938397	G	0.45	0.41	0.16	0.02		**1.17**
**#12**	***SLC39A8***	4	rs13107325	T	0.09	0.07	0.35	0.008		**1.42**
**#13**	***FLJ35779***	5	rs2112347	T	0.62	0.63	−0.07	0.36		*0.93*
**#14**	***NUDT3***	6	rs206936	G	0.23	0.19	0.07	0.44		1.07
**#15**	***TFAP2B***	6	rs987237	G	0.20	0.17	0.22	0.02		**1.24**
**#16**	***LRRN6C***	9	rs10968576	G	0.27	0.31	−0.003	0.97		*0.997*
**#17**	***BDNF (B,M)***	11	rs10767664	A	0.78	0.77	0.16	0.06		1.17
**#18**	***MTCH2***	11	rs3817334	T	0.42	0.41	0.06	0.41		1.06
**#19**	***RPL27A***	11	rs4929949	C	0.46	0.52	−0.06	0.36		*0.94*
**#20**	***FAIM2***	12	rs7138803	A	0.40	0.36	0.21	0.004		**1.23**
**#21**	***MTIF3***	13	rs4771122	G	0.23	0.22	0.09	0.27		1.10
**#22**	***OLFM4***	13	rs9568856	A	0.14	0.12	0.25	0.02		**1.28**
**#23**	***NRXN3***	14	rs10150332	C	0.21	0.21	0.16	0.06		1.18
**#24**	***PRKD1***	14	rs11847697	T	0.06	0.04	0.44	0.007		**1.55**
**#25**	***MAP2K5***	15	rs2241423	G	0.75	0.77	−0.05	0.56		*0.95*
**#26**	***FTO***	16	rs9939609	A	0.49	0.38	0.38	9.2×10^−8^		**1.47**
**#27**	***SH2B1***	16	rs7359397	T	0.33	0.39	−0.14	0.05		*0.87*
**#28**	***HOXB5***	17	rs9299	T	0.64	0.66	−0.09	0.2		*0.91*
**#29**	***MC4R (B)***	18	rs571312	A	0.27	0.22	0.22	0.007		**1.24**
**#30**	***KCTD15***	19	rs29941	G	0.71	0.67	0.17	0.02		**1.19**
**#31**	***QPCTL***	19	rs2287019	C	0.84	0.81	0.16	0.08		1.17
**#32**	***TMEM160 (Q)***	19	rs3810291	A	0.66	0.68	−0.12	0.12		*0.89*

#Ref, reference number of SNPs allocated for Figures 2 and 3; Chr, chromosome; rsid, reference SNP identification number; EA, Effect allele, i.e, BMI-increasing allele as reported in the GIANT-BMI meta-analysis; EAF, effect allele frequency; β; effect size; OR, odds ratio.SNPs yielding at least nominal evidence for association are highlighted in bold and SNPs with effect direction inconsistent with GIANT-BMI results are highlighted in italics.

**Table 3:** Association results with *FTO* SNP rs9939609 and *NEGR1* SNP rs2815752 in categories of BMI, compared with normal-weight controls.

BMI Categories (kg/m^2^)	35.0–39.9	40.0–44.9	45.0–49.9	50.0–59.9	≥60.0
***FTO*** ** SNP**					
**n**	116	237	270	246	84
***P***	0.1	0.01	0.002	0.047	3.4×10^−6^
**β**	0.35	0.35	0.40	0.26	1.08
**SE**	0.21	0.14	0.13	0.13	0.23
***NEGR1*** ** SNP**					
**n**	116	239	266	250	83
***P***	0.11	0.01	0.08	0.07	0.93
**β**	0.32	0.33	0.22	0.22	0.02
**SE**	0.20	0.13	0.12	0.12	0.22

β, effect size; SE, standard error.

**Figure 3:**
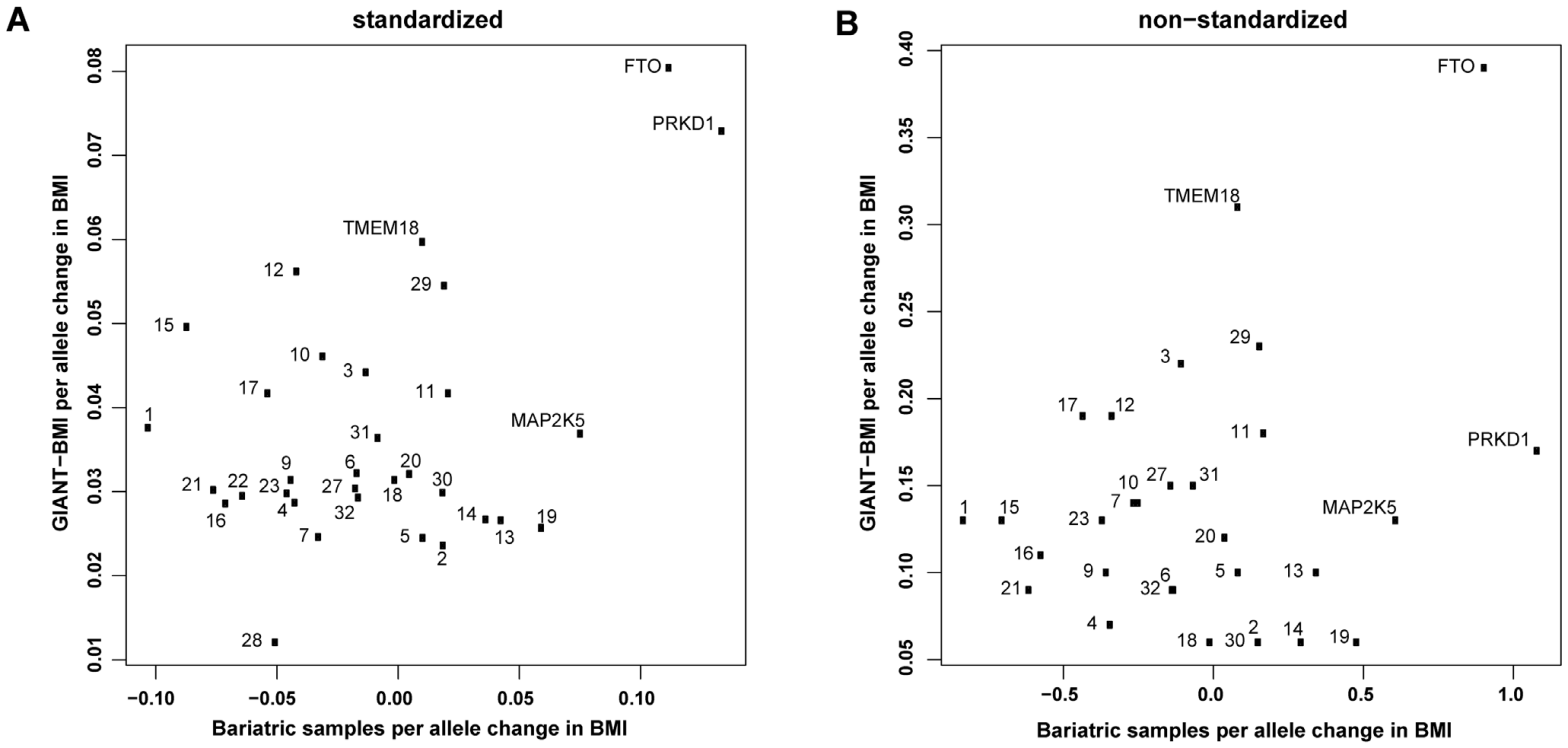
Effect sizes (i.e. changes in BMI) within the bariatric cohort, calculated by using standardized BMI values were compared with the known effect sizes derived from inverse standardized BMI values in the GIANT-BMI meta-analysis [10] (A), and by using unstandardized BMI values (B). Of note, the *FTO* marker effect size plotted for the GIANT-BMI data relates to the SNP rs1558902 (SNP rs9939609 in our study). There were no statistically significant differences between the compared effect sizes. See Table 2 for allocated reference numbers of SNPs.

Next, we examined the association of the BMI-increasing SNPs with BMI within the bariatric surgery cohort alone (n = 967, excluding n = 36 patients who had previous bariatric surgery). Nominal associations with BMI were found only for SNP rs9939609 within the *FTO* gene (*P = *0.01, *β = *0.11±0.04) and with SNP rs2815752 near the *NEGR1* gene, however, paradoxically, there was a negative effect direction for the *NEGR1* locus effect allele (*P = *0.03, *β* = −0.1±0.05). Furthermore, the 32 SNP genetic risk score did not distinguish between BMI values within bariatric surgery patients (*P* = 0.264, adjusted R^2^ = 0.0045). We then undertook linear analyses, using both standardized and unstandardized BMI values, to compare the effects on BMI within the bariatric surgery group with previously published data from the GIANT meta-analysis of adult BMI (GIANT-BMI) [10]. These analyses revealed no significant differences between the compared effect sizes (Figure 3A–B).

## Discussion

In a comparison with normal-weight controls, our analysis of genotype data from European adults with severe obesity attending two bariatric surgery centres has again demonstrated a strong association of the intronic *FTO* SNP rs9939609 with severe obesity. Furthermore, we have shown that a further 13 of the other 31 obesity susceptibility loci that we investigated are at least nominally associated with severe obesity in this cohort of patients being assessed for bariatric surgery. Combination of the 32 SNPs into a genetic risk score convincingly distinguished between normal BMI controls and severely obese patients, which further highlights the influence of common variants on the presence of severe obesity in adults. Our study is, to our knowledge, the first to perform such a comprehensive polygene risk score in a cohort of patients with severe obesity specifically in the setting of a bariatric surgery clinic, ranging from complicated class 2 obesity (BMI 35.0–39.9 kg/m^2^ in the presence of at least one obesity-related comorbidity) to the super super-obese category (BMI ≥60.0 kg/m^2^) (Table 3). Our results suggest that severe obesity represents an extreme of the continuum of BMI variance in the general population, consistent with the results from the recent GIANT-extremes analysis [11].

The methodological approach relating to the polygene risk score represents a novel aspect of our study. While one previous case-control GWAS also specifically studied subjects with severe obesity (mean BMI 50.4±8.1 kg/m^2^), who were attending a bariatric surgery centre, there are important differences between our study design and that of the Cotsapas *et al.* study [16]. After finding a genome-wide association with severe obesity for the *FTO* locus, the investigators then evaluated 12 of the known BMI-associated loci for association with severe obesity. They found that there was a higher burden of risk alleles in patients with severe obesity than in controls and in the more extreme half of BMI distribution within this bariatric cohort [16]. In contrast, we employed a more comprehensive analysis evaluating the contribution of 32 common BMI-increasing SNPs to severe obesity. Rather than using the approach of comparing number of risk alleles as in the Cotsapas *et al.* study, the genetic scores in our study were calculated based on the relative weight of the SNP effect sizes reported in GIANT-BMI meta-analysis. Furthermore, our study employed a comparison with normal-weight controls, whereas the anthropometric data of the controls in the Cotsapas *et al.* study were not available. Previous studies have used such risk scores in the setting of extremes of obesity, with varying results, however these studies employed a more limited panel of SNPs [15], [16], [22], [32]. Nevertheless, the polygenetic approach employed in the GIANT-extremes analysis demonstrates the utility of combining multiple common variants, including those with effect size <0.05, in explaining BMI variance [11].

Our results are consistent with previous studies demonstrating that SNPs in the first intron of *FTO* bear the strongest association with obesity, of the known BMI-raising SNPs [10], [16], [19], and also are strongly associated with extremes of obesity [11], [16], [19], [20], [22], [33], [34]. We found that the *FTO* SNP rs9939609 was also nominally associated with BMI within the severe obese cohort. Furthermore, among the 32 loci, the *FTO* locus held the strongest association and largest effect size in patients with a BMI ≥60.0 kg/m^2^. Taken together, these findings suggest that *FTO* variants retain an important contributory role in the pathogenesis of obesity at increasing levels of severe obesity. The robust association of the *FTO* locus with severe obesity is likely to be mediated through well-documented effects on increasing energy intake [35], and it is highly likely that the altered function in the *FTO* gene itself is mechanistically responsible for the phenotypic effects [36]. However, the mechanisms underlying the *FTO* risk allele phenotype and the SNP effects on *FTO* gene function remain to be fully explored. Interestingly, in this regard, recent evidence suggests that the functional effects of the *FTO* SNP rs9939609 may be mediated through differential methylation of *FTO* itself [37] and myriad other genes [38].

Along with the strong association of the *FTO* locus, we also detected association of the SNP rs2815752 near the *NEGR1* gene with severe obesity. This *NEGR1* SNP ranked among the top four most strongly associated with extremes of obesity of all 32 BMI-associated loci in the GIANT analyses [10], [11]. Interestingly, we found that the *NEGR1* risk allele had a negative effect direction in relation to BMI within the bariatric cohort, a finding that reached nominal significance and in keeping with the consistent decreasing trend in effect size with increasing BMI categories observed (Table 3). Notably, a recent GWAS analysis demonstrated an association of two deletions (43 kb and 8 kb) upstream of *NEGR1* with early onset extreme obesity [22]. Importantly, these deletions segregate on distinct haplotypes [9]. The rs2815752 SNP is known to tag the 43 kb deletion [9], however the protective 8 kb deletion is the major driver of the association with extreme obesity at the *NEGR1* locus and is tagged by an alternative SNP (rs1993709) [22]. In this context, our findings suggest that the *NEGR1* rs2815752 SNP contributes to the genetic risk of severe adult obesity, likely driven by the alternative signal [22], but that the effects may be predominantly relevant at lower points on the continuum of severe adult obesity. The comparable effects observed in our study and data from Wheeler *et al.* [22] highlights the important contribution of the *NEGR1* locus to both adult and early onset forms of severe obesity. However, further studies with increased power are required to confirm our finding that, contrary to the early onset form [22], there is a relatively smaller contribution of the *NEGR1* locus at the extreme tail of the severe adult obesity spectrum. Of note, *NEGR1* has been implicated in hypothalamic control of body weight and food intake [39]. Evidence for a possible functional basis for the association effects of variants at the *NEGR1* locus have also been explored [22]. Evidence that the 8-kb deletion upstream of *NEGR1* encompasses a single binding site for a transcriptional repressor of *NEGR1* begins to provide valuable insights into why these *NEGR1* variants are associated with severe obesity [22], however the downstream mechanisms underlying the association remain to be elucidated.

The lower magnitudes of association with severe obesity found for the other 12 BMI-increasing SNPs suggest that these loci exert a smaller influence on the development of severe obesity. However, power issues relating to the sample size of our study are likely to have impacted upon the strength of the associations. Our finding that only 9 of the remaining 18 SNPs had effects directionally consistent with the GIANT-BMI results [10] raises the possibility that a proportion of SNPs that are associated with BMI in the general population may not contribute to severe obesity. This is in contrast with the data from the GIANT-extremes analysis, in which the effects of all 32 BMI-associated loci on all obesity-related traits were directionally consistent with the prior study of adult BMI, although 4 SNPs were not at least nominally associated with class 3 obesity [11]. However, it is important to note that there is a considerable overlap between the populations used for GIANT-extremes [11] and the prior GIANT-BMI meta-analysis [10]. Our study was undertaken in an independent cohort of patients with severe obesity, and this methodological difference may account for our divergent findings. There is also evidence from the recent study in the SCOOP cohort (UK children of European ancestry with severe early-onset obesity, n = 1,509) [22], that there is an incomplete overlap between loci influencing obesity-related phenotypes among the general adult population (GIANT) or early onset severe obesity (SCOOP). This concept is supported by comparing results from case-control studies of extreme obesity, including our findings, summarized in Table S2 in File S1, suggesting that extreme obesity is a heterogeneous disorder with varying genetic influences, both shared and unshared across the spectrum. Nevertheless, in our study, we did not find any significant differences between odds ratios or effect sizes when compared with GIANT-extremes data [11] (Figure 2) or GIANT-BMI data [10] (Figure 3A–B) respectively. Therefore, the relatively small sample sizes in the studies summarized in Table S2 in File S1 may have impacted upon the strength of the associations with common BMI-associated variants detected, in particular for risk alleles with relatively lower frequencies such as the *PRKD1* risk allele. Interestingly, the sample size in our study compares well with that of class 3 obesity in the GIANT-extremes study, drawn from a pool of over 260,000 individuals, highlighting the productive potential for undertaking genetic studies in patients attending bariatric centres.

There are a number of potential limitations pertaining to our study, chief amongst them is the lack of a ‘hypothesis-free’ study design. Our results should be interpreted with caution, in this regard, as our research question may have introduced a bias into the findings. Furthermore, our study did not address other genetic factors such as highly penetrant rare variants, that may exert an increasing contribution in more extreme obesity and therefore contribute to the ‘missing heritability’ of BMI-related phenotypes [12]. For example, the recent genome-wide copy number variation (CNV) analysis again in the SCOOP cohort demonstrated a higher burden of rare, and in particular, singleton CNVs in the extreme obesity cohort compared to controls [22]. Furthermore, we acknowledge that our study is insufficiently powered to replicate findings for all BMI-associated loci, many of which were identified only using sample sizes several orders of magnitude higher than in our study [10]. However, the potential to replicate some of the strongest signals remained and we were also able to test if any known loci had stronger effects in such an extreme obesity dataset compared to the published population-based data.

Our findings in relation to the modest effects of these specific common BMI-associated variants, as aptly demonstrated in Figure 1, are consistent with the well-documented gap between explained variance due to common variants (∼2%) and estimated heritability (h^2^) of obesity (∼40–70%) [12], [13], [40]. However, a novel approach called genome-wide complex trait analysis (GCTA) has yielded results that suggest there are a multitude of low penetrance common variants, each with causal effects too small to allow detection by GWAS, together accounting for up to 17% of the overall BMI variance [41], which has been further corroborated by the GIANT-extremes polygene analysis [11]. Such a GCTA approach has also been undertaken in a recent analysis of twin studies and revealed that 37% of BMI h^2^ could be explained by the effects of multiple common SNPs [42]. An additional consideration is that the heritability of severe obesity is not as well delineated as for overweight and lower levels of obesity, although familial aggregation of severe obesity is well documented [40]. Many of the classical twin studies involve less obese populations and are not directly generalizable to severe obesity [7], [8]. Gene-environment interactions are another potential explanation for the unexplained heritability [40]. In this light, while our results suggest that accumulation of common variants predisposes to severe obesity, actual BMI in adults with severe obesity may be relatively more dictated by other factors including environmental influences [43], compared to individuals in lower BMI categories.

In summary, we have demonstrated that, among 32 BMI-increasing common variants, at least 2 are strongly associated and 12 other variants are nominally associated with severe obesity in patients attending a bariatric surgery centre. Combination of all 32 genotyped SNPs in a genetic risk score was associated with severe obesity, however the risk score was not associated with actual BMI within the bariatric cohort. We conclude that significant effects of individual BMI-associated common variants can be found even in a relatively small sample size, in a comparison of a bariatric cohort to normal-weight controls, and that the burden of such low-penetrant risk alleles contributes to severe obesity in this population. These findings add more support to the hypothesis that severe obesity represents an extreme tail of the population BMI variation. However, the limitations of our study prevent us from drawing any conclusions regarding the relative importance of common genetic variants compared to other factors, genetic or otherwise, that are likely to contribute to severe obesity. Nevertheless, future genetic studies focused on bariatric patients may provide valuable insights into the pathogenesis of obesity at a population level.

## Supporting Information

File S1 Model S1. Formula for standardization of BMI values. Model S2. Model used for calculation of genetic risk score. Table S1. Comparison of additive, dominant and recessive models for logistic regression analysis. Table S2. Comparison of case-control analysis results (odds ratios) in 6 cohorts of extreme obesity for common BMI-associated loci.
(DOCX)
