# Supplementary material for: Contribution of 32 GWAS-Identified Common Variants to Severe Obesity in European Adults Referred for Bariatric Surgery
Source: PLoS One. 2013 Aug 7;8(8):e70735. doi: 10.1371/journal.pone.0070735 (PMC3737377; doi:10.1371/journal.pone.0070735)
Supplement: File S1 — Model S1. Formula for standardization of BMI values. Model S2. Model used for calculation of genetic risk score. Table S1. Comparison of additive, dominant and recessive models for logistic regression analysis. Table S2. Comparison of case-control analysis results (odds ratios) in 6 cohorts of extreme obesity for common BMI-associated loci. (DOCX) [file pone.0070735.s001.docx]

**Supporting Information (SI)**

**“Contribution of 32 GWAS-identified common variants to severe obesity in European adults referred for bariatric surgery”**

**Model S1.** Formula for standardization of BMI values

The standard score of a raw score x is z = (x-μ)/δ, where μ is the mean of the population and σ is the standard deviation of the population.

**Model S2.** Model used for calculation of genetic risk score

((rs1558902:A x 0.39) + (rs2867125:C x 0.31) + (rs571312:A x 0.23) + (rs10938397:G x 0.18) + (rs10767664:A x 0.19) + (rs2815752:A x 0.13) + (rs7359397:T x 0.15) + (rs9816226:T x 0.14) + (rs3817334:T x 0.06) + (rs29941:G x 0.06) + (rs543874:G x 0.22) + (rs987237:G x 0.13) + (rs7138803:A x 0.12) + (rs10150332:C x 0.13) + (rs713586:C x 0.14) + (rs12444979:C x 0.17) + (rs2241423:G x 0.13) + (rs2287019:C x 0.15) + (rs1514175:A x 0.07) + (rs13107325:T x 0.19) + (rs2112347:T x 0.10) + (rs10968576:G x 0.11) + (rs3810291:A x 0.09) + (rs887912:T x 0.10) + (rs13078807:G x 0.10) + (rs11847697:T x 0.17) + (rs2890652:C x 0.09) + (rs1555543:C x 0.06) + (rs4771122:G x 0.09) + (rs4836133:A x 0.07) + (rs4929949:C x 0.06) + (rs206936:G x 0.06))/(32*callrate)

For each sample the sum of all BMI rising alleles weighted by their effect size, as reported in the GIANT-BMI meta-analysis, is calculated and then divided by the number of non-missing SNPs.

**Table S1.** Comparison of additive, dominant and recessive models for logistic regression analysis

| Gene | rsid | Effect allele |  | | | Models | | |  | | |
| --- | --- | --- | --- | --- | --- | --- | --- | --- | --- | --- | --- |
|  |  |  | **Additive** | | | **Dominant** | | | **Recessive** | | |
|  |  |  | ***P*** | **β** | **SE** | ***P*** | **β** | **SE** | ***P*** | **β** | **SE** |
| *NEGR1* | rs2815752 | A | 3.6x10^-4^ | 0.25 | 0.07 | 0.01 | 0.42 | 0.13 | 0.001 | 0.28 | 0.10 |
| *PTBP2* | rs1555543 | C | 0.08 | -0.12 | 0.07 | 0.04 | -0.08 | 0.13 | 0.52 | -0.21 | 0.10 |
| *SEC16B* | rs543874 | G | 0.04 | 0.17 | 0.08 | 0.27 | 0.20 | 0.10 | 0.05 | 0.26 | 0.24 |
| *TNNI3K* | rs1514175 | G | 0.01 | 0.18 | 0.07 | 0.07 | 0.24 | 0.10 | 0.02 | 0.23 | 0.13 |
| *FANCL* | rs887912 | C | 0.09 | 0.13 | 0.08 | 0.08 | 0.12 | 0.10 | 0.22 | 0.31 | 0.17 |
| *LRP1B* | rs2890652 | C | 0.02 | 0.23 | 0.10 | 0.01 | 0.20 | 0.11 | 0.07 | 0.83 | 0.32 |
| *RBJ* | rs713586 | C | 0.95 | -0.004 | 0.07 | 0.41 | -0.10 | 0.11 | 0.36 | 0.09 | 0.11 |
| *TMEM18* | rs2867125 | C | 0.005 | 0.26 | 0.09 | 0.003 | 0.20 | 0.29 | 0.50 | 0.31 | 0.10 |
| *CADM2* | rs13078807 | G | 0.11 | 0.14 | 0.09 | 0.32 | 0.15 | 0.10 | 0.13 | 0.26 | 0.26 |
| *ETV5* | rs9816226 | T | 0.07 | 0.17 | 0.09 | 0.03 | -0.07 | 0.28 | 0.79 | 0.23 | 0.10 |
| *GNPDA2* | rs10938397 | G | 0.02 | 0.16 | 0.07 | 0.21 | 0.24 | 0.10 | 0.02 | 0.16 | 0.13 |
| *SLC39A8* | rs13107325 | T | 0.008 | 0.35 | 0.13 | 0.16 | 0.36 | 0.14 | 0.01 | 0.85 | 0.61 |
| *FLJ35779* | rs2112347 | G | 0.36 | -0.07 | 0.07 | 0.65 | -0.17 | 0.14 | 0.24 | -0.05 | 0.10 |
| *NUDT3* | rs206936 | G | 0.44 | 0.07 | 0.09 | 0.22 | 0.04 | 0.10 | 0.69 | 0.29 | 0.24 |
| *TFAP2B* | rs987237 | G | 0.02 | 0.22 | 0.09 | 0.0001 | 0.14 | 0.10 | 0.19 | 1.18 | 0.31 |
| *LRRN6C* | rs10968576 | G | 0.97 | -0.003 | 0.07 | 0.45 | -0.05 | 0.10 | 0.61 | 0.13 | 0.16 |
| *BDNF (B,M)* | rs10767664 | A | 0.06 | 0.16 | 0.08 | 0.04 | 0.15 | 0.23 | 0.51 | 0.20 | 0.10 |
| *MTCH2* | rs3817334 | T | 0.41 | 0.06 | 0.07 | 0.91 | 0.13 | 0.10 | 0.19 | -0.02 | 0.13 |
| *RPL27A* | rs4929949 | C | 0.36 | -0.06 | 0.07 | 0.66 | -0.12 | 0.11 | 0.27 | -0.05 | 0.11 |
| *FAIM2* | rs7138803 | A | 0.004 | 0.21 | 0.07 | 0.001 | 0.18 | 0.10 | 0.07 | 0.45 | 0.14 |
| *MTIF3* | rs4771122 | A | 0.27 | 0.09 | 0.08 | 0.58 | 0.11 | 0.10 | 0.29 | 0.13 | 0.23 |
| *OLFM4* | rs9568856 | A | 0.02 | 0.25 | 0.10 | 0.19 | 0.27 | 0.11 | 0.02 | 0.47 | 0.36 |
| *NRXN3* | rs10150332 | C | 0.06 | 0.16 | 0.08 | 0.02 | 0.13 | 0.10 | 0.21 | 0.56 | 0.24 |
| *PRKD1* | rs11847697 | T | 0.007 | 0.44 | 0.16 | 0.25 | 0.45 | 0.17 | 0.01 | 1.01 | 0.89 |
| *MAP2K5* | rs2241423 | A | 0.56 | -0.05 | 0.08 | 0.40 | -0.07 | 0.22 | 0.74 | -0.08 | 0.10 |
| *FTO* | rs9939609 | A | 9.2x10^-8^ | 0.38 | 0.07 | 1.7x10^-7^ | 0.38 | 0.10 | 0.0002 | 0.71 | 0.14 |
| *SH2B1* | rs7359397 | T | 0.05 | -0.14 | 0.07 | 0.22 | -0.19 | 0.10 | 0.06 | -0.17 | 0.14 |
| *HOXB5* | rs9299 | T | 0.2 | -0.09 | 0.07 | 0.53 | -0.25 | 0.15 | 0.09 | -0.06 | 0.10 |
| *MC4R (B)* | rs571312 | A | 0.007 | 0.22 | 0.08 | 0.02 | 0.21 | 0.10 | 0.03 | 0.50 | 0.21 |
| *KCTD15* | rs29941 | G | 0.02 | 0.17 | 0.07 | 0.13 | 0.41 | 0.16 | 0.01 | 0.15 | 0.10 |
| *QPCTL* | rs2287019 | T | 0.08 | 0.16 | 0.09 | 0.13 | 0.39 | 0.29 | 0.17 | 0.16 | 0.10 |
| *TMEM160 (Q)* | rs3810291 | A | 0.12 | -0.12 | 0.07 | 0.14 | -0.16 | 0.16 | 0.32 | -0.15 | 0.10 |

rsid, reference SNP identification number; Effect allele, BMI-increasing allele as reported in the GIANT-BMI meta-analysis β, effect size; SE, standard error

**Table S2.** Comparison of case-control analysis results (odds ratios) in 6 cohorts of extreme obesity for common BMI-associated loci

| Loci | Effect allele^a^ | Bariatric^b^ | GIANT-extremes^c^ | | SCOOP^d^ | Cotsapas et al.^e^ | | Wang et al.^f^ | Zhao et al.^g^ |
| --- | --- | --- | --- | --- | --- | --- | --- | --- | --- |
| *NEGR1* | A | **1.29** | **1.19** | **1.25** | | **1.11^h^** | 0.96 | | 1.01^h^ |
| *PTBP2* | C | *0.88* | **1.08** | 1.04 | | - | 0.96 | | 1.06^h^ |
| *SEC16B* | G | **1.19** | **1.23** | **1.22** | | 1.07 | 1.11 | | **1.21** |
| *TNNI3K* | A | **1.19** | 1.06 | **1.19** | | - | 1.07 | | **1.14** |
| *FANCL* | T | 1.14 | **1.13** | 1.00 | | - | **1.39** | | *0.98* |
| *LRP1B* | C | **1.26** | **1.17** | **1.23** | | - | 1.12 | | 1.08 |
| *RBJ* | C | *0.996* | **1.10** | 1.07 | | - | 1.09 | | 1.11 |
| *TMEM18* | C | **1.29** | **1.25** | **1.39** | | **1.18^h^** | 0.84 | | **1.28^h^** |
| *CADM2* | G | 1.15 | 1.08 | 1.04 | | - | 1.22 | | 1.06 |
| *ETV5* | T | 1.18 | **1.15** | **1.16** | | *0.86* | 0.82 | | *0.996^h^* |
| *GNPDA2* | G | **1.17** | **1.20** | **1.21** | | **1.18** | 1.03 | | **1.14** |
| *SLC39A8* | T | **1.42** | **1.29** | - | | - | 0.99 | | 1.07 |
| *FLJ35779* | T | *0.93* | **1.11** | 1.06 | | - | 0.98 | | *0.98^h^* |
| *NUDT3* | G | 1.07 | **1.09** | 1.02 | | - | 1.006 | | 1.07 |
| *TFAP2B* | G | **1.24** | **1.29** | **1.14** | | - | 1.20 | | 1.1 |
| *LRRN6C* | G | *0.997* | **1.12** | 1.04 | | - | 0.97 | | 1.02 |
| *BDNF(B,M)* | A | 1.17 | **1.10** | **1.19** | | 1.13 | 0.85 | | **1.14^h^** |
| *MTCH2* | T | 1.06 | **1.09** | 1.02 | | 1.09 | 1.12 | | *0.97* |
| *RPL27A* | C | *0.94* | **1.09** | 1.04 | | 1.09 | 1.02 | | 1.05^h^ |
| *FAIM2* | A | **1.23** | **1.17** | **1.23** | | - | 1.10 | | 1.08 |
| *MTIF3* | G | 1.1 | **1.10** | - | | - | 0.91 | | 1.11 |
| *OLFM4*^i^ | A | **1.28** | 1.08 | **1.15** | | - | - | | - |
| *NRXN3* | C | 1.18 | **1.12** | 1.04 | | - | 1.11 | | **1.23** |
| *PRKD1* | T | **1.55** | 1.01 | **1.30** | | - | 1.07 | | *0.98* |
| *MAP2K5* | G | *0.95* | **1.13** | **1.13** | | - | **0.73** | | 1.03^h^ |
| *FTO* | A | **1.47** | **1.45** | **1.48** | | **1.46** | **1.63** | | **1.34** |
| *SH2B1* | T | *0.87* | **1.08** | 1.07 | | **1.12** | 1.18 | | 1.05 |
| *HOXB5*^i^ | T | *0.91* | 1.01 | - | | - | - | | - |
| *MC4R(B)* | A | **1.24** | **1.21** | **1.32** | | 1.02 | **1.26** | | **1.21** |
| *KCTD15* | G | **1.19** | **1.1** | 1.07 | | **1.15^h^** | **0.81** | | 1.09^h^ |
| *QPCTL* | C | 1.17 | **1.22** | - | | - | 0.80 | | **1.18^h^** |
| *TMEM160(Q)* | A | *0.89* | **1.12** | - | | - | 0.90 | | 1.04^h^ |
| *ZNF608*^j^ | A | - | 1.06 | - | | - | - | | 1.10^h^ |
| *GPRC5B*^j^ | C | - | **1.19** | 1.10 | | - | **0.74** | | 1.03^h^ |

^a^Effect allele, BMI-increasing allele for SNPs as reported in the GIANT-BMI meta-analysis; ^b^Bariatric, our case-control analysis in European adults with severe obesity attending bariatric clinics (n=1,003); ^c^GIANT-extremes, obesity class 3 (n = 3,892) case-control results of meta-analysis of population-based GWAS;  ^d^SCOOP, GWAS in children of European ancestry with severe early-onset obesity (n = 1,509); ^e^Cotsapas et al., GWAS in Caucasian adults with severe obesity (n = 775), attending a bariatric clinic, results were reported for 12 known common BMI-associated loci as study was pre-GIANT-BMI meta-analysis, included results based on best proxy SNPs; ^f^Wang et al., GWAS in Caucasian adults with severe obesity (n = 520), included results based on best proxy SNPs, effect direction for BMI-increasing alleles was not reported, thus evidence for directional consistency was not shown; ^g^Zhao et al., case-control analysis in European American children with obesity (n = 1,097); ^h^results corrected for BMI-increasing allele in place of results for allele reported; ^i^new loci uncovered in meta-analysis of childhood obesity; ^j^SNPs not available for analysis in our study. SNPs yielding at least nominal evidence for association are highlighted in bold. SNPs with effects directionally inconsistent with GIANT meta-analysis of adult BMI are highlighted in italics.
